# Recent Trends in Non-invasive Neural Recording Based Brain-to-Brain Synchrony Analysis on Multidisciplinary Human Interactions for Understanding Brain Dynamics: A Systematic Review

**DOI:** 10.3389/fncom.2022.875282

**Published:** 2022-06-16

**Authors:** Tahnia Nazneen, Iffath Binta Islam, Md. Sakibur Rahman Sajal, Wasifa Jamal, M. Ashraful Amin, Ravi Vaidyanathan, Tom Chau, Khondaker A. Mamun

**Affiliations:** ^1^Advanced Intelligent Multidisciplinary Systems Lab, Institute of Advanced Research, United International University, Dhaka, Bangladesh; ^2^Department of Computer Science and Engineering, United International University, Dhaka, Bangladesh; ^3^BrainCo Inc., Somerville, MA, United States; ^4^Department of Computer Science and Engineering, Independent University, Dhaka, Bangladesh; ^5^Department of Mechanical Engineering, Imperial College London, London, United Kingdom; ^6^Institute of Biomaterials and Biomedical Engineering, University of Toronto, Toronto, ON, Canada

**Keywords:** behavioral cognition, brain dynamics, brain-to-brain synchrony, interbrain synchrony, neuroscience, psychology, synchrony measure

## Abstract

The study of brain-to-brain synchrony has a burgeoning application in the brain-computer interface (BCI) research, offering valuable insights into the neural underpinnings of interacting human brains using numerous neural recording technologies. The area allows exploring the commonality of brain dynamics by evaluating the neural synchronization among a group of people performing a specified task. The growing number of publications on brain-to-brain synchrony inspired the authors to conduct a systematic review using the PRISMA protocol so that future researchers can get a comprehensive understanding of the paradigms, methodologies, translational algorithms, and challenges in the area of brain-to-brain synchrony research. This review has gone through a systematic search with a specified search string and selected some articles based on pre-specified eligibility criteria. The findings from the review revealed that most of the articles have followed the social psychology paradigm, while 36% of the selected studies have an application in cognitive neuroscience. The most applied approach to determine neural connectivity is a coherence measure utilizing phase-locking value (PLV) in the EEG studies, followed by wavelet transform coherence (WTC) in all of the fNIRS studies. While most of the experiments have control experiments as a part of their setup, a small number implemented algorithmic control, and only one study had interventional or a stimulus-induced control experiment to limit spurious synchronization. Hence, to the best of the authors' knowledge, this systematic review solely contributes to critically evaluating the scopes and technological advances of brain-to-brain synchrony to allow this discipline to produce more effective research outcomes in the remote future.

## 1. Introduction

In an attempt to explicate the presence of unusually large brain size in primates, the social brain hypothesis was first proposed by Dunbar (1998), postulating that primate brains developed certain brain regions to accommodate higher-order conspecific interactions. Additionally, the correlation between human interactions and the perceived identical patterns of the human body's oscillatory physiological activities (e.g., heart rate or breathing or footsteps) were acknowledged by social and cognitive neuroscience researchers alike (Zhang and Zhang, 2018). However, due to the advancement and development of neural signal recording technologies, these behavioral and psychological synchronies were also been observed through a multitude of experiments in domains of neuroscience and psychology by measuring and analyzing electrical, magnetic, and hemodynamic activities of the brain. The increased availability of both research and industry-grade non-invasive neural signal recording devices further led to the acquisition and analysis of the neural signal from multiple brains simultaneously. This approach, commonly known as hyperscanning (Astolfi et al., 2011), allowed researchers to comprehensively examine the synchronization of two or more brains participating in the same activity and infer the effect of these synchronizations on behavioral performance in the field of brain computer interface (BCI). Thus, the study of brain-to-brain synchrony constructed an interdisciplinary bridge between neuroscientists and psychologists to reveal the ubiquity of synchronized human brains and their underlying neurobiological elements and functionalities in great detail in BCI (Dikker et al., 2017; Bevilacqua et al., 2019; Davidesco et al., 2019).

After the first experiment conducted to study interpersonal neural synchronization using fMRI in 2002 (Montague, 2002), the field of brain-to-brain synchrony has only seen progress with the introduction of both novel and adapted paradigms, methods, and modalities in each study. Multibrain neural activity is now being recorded through non-invasive neuroimaging methods with unprecedented details helping the researchers to analyze the data in BCI research in a sophisticated manner. The neuroimaging methods that have been used to analyze more than one brain simultaneously are electroencephalogram (EEG), functional near-infrared spectroscopy (fNIRS), functional magnetic resonance imaging (fMRI), etc. These non-invasive neural recording techniques have been preferable to researchers due to their risk-free application and easy to use on healthy participants to study the cognitive function in a sterile laboratory environment or naturalistic settings.

Over the last decades, researchers experimenting on the social human brain recorded and measured the neural connectivities by adopting hyper-scanning techniques in designing and monitoring several host of tasks, such as prisoners dilemma game (Jahng et al., 2017; Hu et al., 2018), guitar playing (Sänger et al., 2012), interaction in a virtual environment (Gumilar et al., 2021), debate (van Vugt et al., 2020), video watching (Ding et al., 2018; Gao et al., 2020), parent-child dyad (Reindl et al., 2018; Santamaria et al., 2019), playing video games (Liu et al., 2016), etc. These studies essentially upgraded and replaced the traditional study of the individual brain in isolation with the study interactive nature of human cognition as an alternative. Dikker et al. predicted students' engagement in the classroom and Dana Bevilacqua et al. predicted retention memory, learning outcome, and the relation between student and teacher in the classroom as naturalistic settings using the method of total interdependence (Dikker et al., 2017). Another most prominent social interaction is verbal interaction between individuals. The study of Alejandro Perez et al. has described the verbal exchange of two persons by turn-taking using brain-to-brain phase synchrony without visual contact from scalp topography (Pérez et al., 2019). To keep pace with the increasing number of paradigms and field of studies in BCI explored for each signal acquisition modality for studying brain-to-brain synchrony, various intra-brain synchrony measures were continuously being updated and utilized to correlate between behavioral and neural activities in groups. These progress in the domains, modalities, methodologies, and experimental controls in the ever-growing number of studies call for an explicit and structured record of the issues that prevail in current studies following their analysis and critical appraisal. However, the 25 reviews (as of the search conducted on 22 December 2020) on brain-to-brain synchrony have been narrative in nature, assessing only certain aspects of the field, displaying notable limitations in methods, contents, and approaches. The only systematic review published in this field (Nam et al., 2020) covers the neuroimaging method, a relatively small application domain, and the experimental paradigm, leaving out the various synchrony measures and the analysis of the epiphenomenon hypothesis of brain-to-brain synchrony. Hence, we feel an urge to drive another systematic review covering the methodological part of analyzing brain-to-brain synchrony. For this reason, we have highlighted the relation of modalities with paradigms of study, measuring techniques and translational algorithm, influential internal and external factors while measuring neural synchrony and give brief reasoning about better modalities in respective perspectives. Owing to the development of inter-brain synchrony measures and portable devices, larger group sizes and a wider range of behavioral studies have been observed in the past decade. This necessitated a more comprehensive review following the Preferred Reporting Items for Systematic Review and Meta-Analysis Protocol (PRISMA-P) to address four specific research questions. The questions (RQs) with the respective scopes and rationale used to evaluate them are listed in Table 1.

**Table 1:** Research questions (RQs) addressed in the systematic review.

**RQ#**	**Research questions**	**Scopes**	**Rationale**
RQ1	What domains are explored in the analysis of brain-to-brain synchrony in the selected studies?	The domain of neuroscience and psychological paradigm has been explored to define the field of study and experimental design for measuring brain-to-brain synchrony, along with assigned tasks and significant outcomes of the studies.	This is an exhaustive question seeking to explore the entire scope of brain-to-brain synchrony research. An overarching study of its domains will help future researchers group their studies and design newer paradigms.
RQ2	What modalities are used to collect neural signals from the brain and why?	The types of neural signals (EEG/MEG, fMRI, fNIRS, etc.) and the respected devices to collect this neural signal are tabulated. The reason for choosing the respected neural signal has also been described.	This is the most important aspect when analyzing and correlating studies with the environmental setting and neural synchrony measure.
RQ3	What neural synchrony measures are used?	The preprocessing method, connectivity measure, behavioral and neural correlation, and directional measures are extracted and analyzed.	The methods or indices that are used for measuring brain-to-brain synchrony can give the researchers an insight into the evolution and current limitation of the algorithms.
RQ4	What internal and external factors have influenced the inter-bain synchrony?	This brings into light the study of spurious synchronization and current limitations in measurement techniques	The details and methods to differentiate spurious synchrony from true synchrony can guide future researchers in designing more robust control and interventional experiments.

In this systematic review, neural synchrony among people has been analyzed using various linear and non-linear methods in a large number of recent publications where the authors have used the term brain-to-brain synchrony with similar kinds of affinities like interbrain synchrony (Hu et al., 2018), interpersonal neural synchronization (Novembre et al., 2017), or phase synchrony (Poikonen et al., 2018), etc. to determine neural synchronization among a group of participants.

The organization of this systematic review has followed the following procedure. First, it has been organized with a brief introduction of conceptual description and documentation about brain-to-brain synchrony and the research questions are also developed to give a comprehensive idea to future researchers. Second, a searching strategy and criterion have been developed to select the studies that were synthesized and analyzed. Next, the research questions are critically analyzed with the collected data from the screened table, and the analysis from the selected studies is provided in the result section. Later, the systematic review delivers a brief discussion on the perspective of the analysis and finally, concludes the review.

## 2. Methodology

A systematic review is a process to summarize the relevant articles and studies for evaluating one or more research questions maintaining specific eligibility criteria and protocol for screening, selection, and synthesizing data (PRISMA-P Group et al., 2015). In this systematic review, journals, conference papers, and electronic pre-print published in the English language between 2011 and 2020 from the popular databases have been selected. The reason behind selecting the articles of the last decades was to observe cutting-edge trends that are used for evaluating neural synchrony using BCI technology. The trend of witnessing traits of the human brain for cognitive behavior and social interaction while doing similar activity has been aggregating over the last decade. Hence, the authors have decided to conduct a systematic review focusing to evaluate current techniques for various synchrony measures and the analysis of modern development while analyzing brain-to-brain synchrony from the scanning of the human brain for participating in the same task. The databases used here are Google Scholar, Pubmed, bioRxiv, and Science Direct. BioRxiv is a preprint server where non-peer-reviewed articles have been published. The studies from the electronic preprints database are important because of its prompt release cycle. Additionally, searching through these preprints minimize the possibility of biased work and allow us to enhance the diversity of the searching area for a rigid review (Paez, 2017).

A search string has been generated for queries in the databases. Relevant studies have been selected by screening the abstracts and titles thoroughly from these databases. This systematic review includes some additional relevant studies from screening the bibliography of the selected articles. The search string and the number of articles collected from the corresponding databases are given below in Table 2. The authors carried out the final query of the strings on selected databases on 22 November, 2020.

**Table 2:** Searching Strategy from the databases with a multidisciplinary perspective.

**Database**	**Search string**	**Records identified**	**Perspective**
Google scholar	“brain-to-brain” OR neural OR interbrain AND synchron* OR coupling OR hyperscan*	300	Cross-disciplinary perspective
Pubmed	(“brain-to-brain” OR neural OR interbrain OR hyperscan*) AND (synchron* OR coupling)	136	Medical perspective
Science direct	“brain-to-brain” OR neural OR interbrain AND synchron* OR coupling	261	Cross-disciplinary perspective
IEEE Xplore	“brain-to-brain” OR neural OR interbrain AND synchron* OR coupling	9	Electrical/electronic engineering Perspective
Web of science	“brain-to-brain” OR neural OR interbrain AND synchron* OR coupling	199	Cross-disciplinary perspective
bioRxiv	“brain-to-brain” OR neural OR interbrain AND synchron* OR coupling	86	Neuroscience and physiology perspective
		Total = 991	

**Figure 1:**
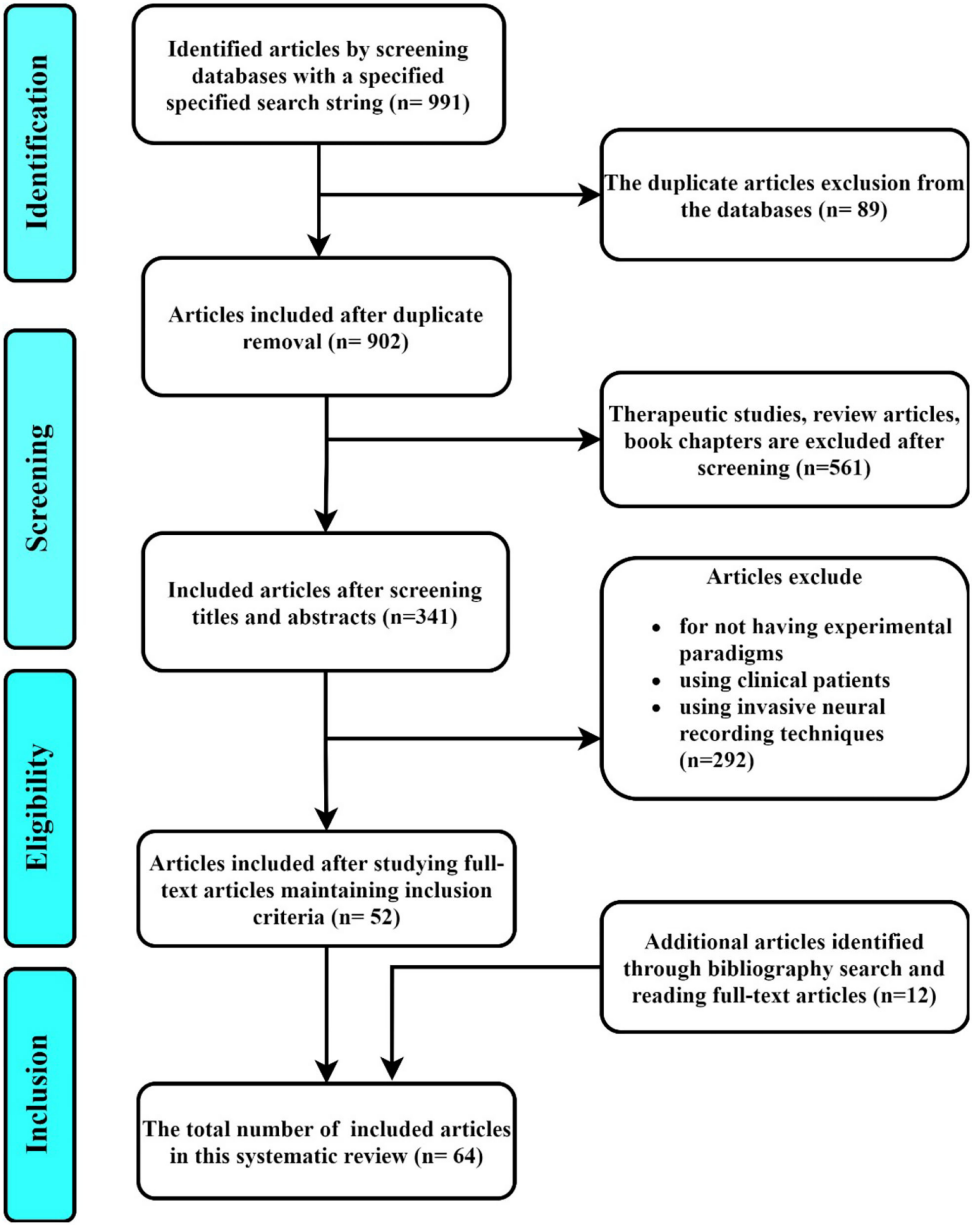
Flow chart for selection of studies maintaining Preferred Reporting Items for Systematic Review and Meta-Analysis Protocol (PRISMA-P) protocol.

The first screening has identified 991 articles that included the keywords in the search string either in their title, abstract, full-text, or metadata. The authors have organized a meeting to decide the inclusion and exclusion criteria according to PRISMA-P (Preferred Reporting Items for Systematic Review and Meta-Analysis Protocol) protocol (Welch et al., 2012; Shamseer et al., 2015). The decided inclusion and exclusion criteria (showed in Table 3) pave us to identify the most relevant articles from the selected databases focusing to evaluate the research questions more strongly. Two reviewers have gone through the full-text articles of these selected studies to narrow down the selection based on inclusion and exclusion criteria. Finally, the whole process leads us to 64 significant articles that contain all the necessary information to evaluate the research questions maintaining the eligibility criteria at our best possible attempt. The whole process has been shown by a flowchart maintaining the PRISMA-P protocol in the Figure 1. For evaluating the research questions, each author has built a table, two authors have blindly screened the final 64 articles to synthesize the data. Then, they merged their tables to scrutinize the right set of information during synthes of the data from the selected studies. This inspection helped to create a final table containing the imperative set of synthesized data. This table has been provided to the other authors to recheck the information. The table has been reformed and refined according to their valuable advice. This organized table has been created by following the PRISMA-P checklist recommended for systematic review (PRISMA-P Group et al., 2015). This organized table has been created by following the PRISMA-P checklist recommended for the systematic review. The table has been shown in Table 4 containing 20 data items. These data items are categorized into seven types: the origin of articles, domains, data acquisition, neural signal processing, control condition, outcomes, and reproducibility. Each category contains a set of data items to evaluate the research questions. The entire table can be found aimsl.uiu.ac.bd.

**Table 3:** Inclusion and exclusion criteria of the systematic review.

**Inclusion critera**	**Exclusion critera**
Literatures that have been published from 2011 to 2020. Studies having an experimental paradigm in naturalistic or laboratory settings. Studies published only in the English language are included. Accepted manuscripts and pre-print versions of studies are also eligible for inclusion criteria.	Therapeutic studies, review articles, book chapters are excluded from this study. Studies using animals for experimental paradigms have been excluded. Clinical patients (participants with any health issue or mental disorder) and clinical research are also excluded from this article.

**Table 4:** Extracted data items for the selected articles.

**Category**	**Data item**	**Description**
Origin of articles	Type of paper	The publication of studies as an article of journal or conference paper or in the repository of electronic preprint.
	Country of Affiliation of the first author	The location of the affiliated institute, university, or research lab of the first author
	Year	The year of publication of the article
Domain	Experimental Paradigm	Experimental design followed by the study in terms of participants' role.
	Environment	Whether or not the study was conducted in a naturalistic or laboratory environment
	Tasks	The list of tasks were given to the participants whose associated neural synchrony was measured
	Field of study	Primary area of study of the selected experiment or study
Data acquisition	Modality	Whether the signal derivation was based on electrical, hemodynamic, or blood flow activity of the brain
	Hardware	If the type of hardware used in the study was research-grade or commercial-grade
Neural signal processing	Preprocessing	Set of manipulation steps applied to the raw data to remove artifact and noise
	Synchrony measure	Synchrony measures that were used to measure the synchrony index
Control condition	Induced Synchrony	Whether or not there was a procedure in place to eliminate the possibility of synchrony due to shared stimuli perception, if so, which
	Coincidence synchrony	Whether or not there was a procedure in place to rule out the possibility of coincidental synchrony, if so, which
Outcomes	Behavior and synchrony	Dynamic effect of the mutual task on human brain signal
	Activated brain region	The brain regions that were activated in the selected studies
	Synchronized frequency	The frequency level at which the interbrain synchronization signified true information flow among brains
Reproducibility	Dataset	Whether the data used for the experiment has a publicly available dataset or not, and if so, where
	Code	Whether the code used for the experiment is available online or not, and if so, where

## 3. Results

The database queries returned 991 results that matched the search items. Upon intensively screening through individual studies' title, abstract, keywords, and scope of the search results, and following the removal of duplicates from the results, 52 papers proceeded to the eligibility screening stage. The researchers then identified 12 additional papers through a bibliographic search of the selected articles. Sixty four studies met the eligibility criteria and were selected for critical review and analysis.

### 3.1. Origin of the Selected Studies

**Figure 2:**
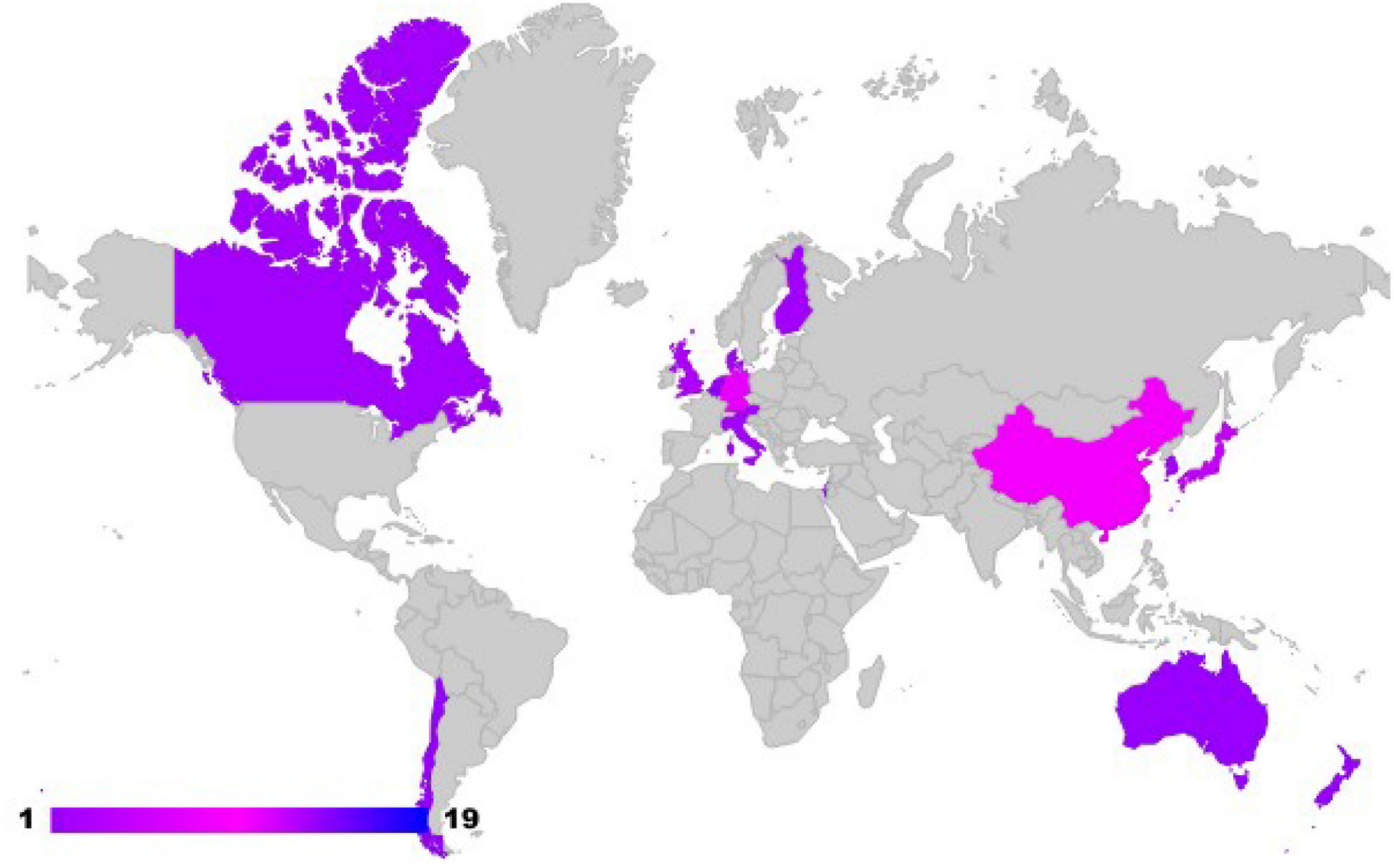
A number of studies conducted in different countries according to first author affiliation.

The search methodology returned 56 journal articles, 3 conference papers, and 5 preprints. A surprising 13 articles (20.31%) were published in *NeuroImage*, followed by *Scientific Reports* and *Social Cognitive and Affective Neuroscience* publishing 6 (9.37%) articles each. Additionally, the researchers looked at the country of affiliation of the first author to identify the geographical distribution of research in the field of interbrain synchrony research, represented in Figure 2. Twenty two or 34.37% of the selected studies were conducted in laboratories and universities situated in the United States with the rest of them mostly being clustered in countries such as China (9 or 14.06%), Germany (8 or 12.50%), Japan (5 or 7.81%), and United Kingdom (3 or 4.68%), and so on.

### 3.2. Year of Publications

**Figure 3:**
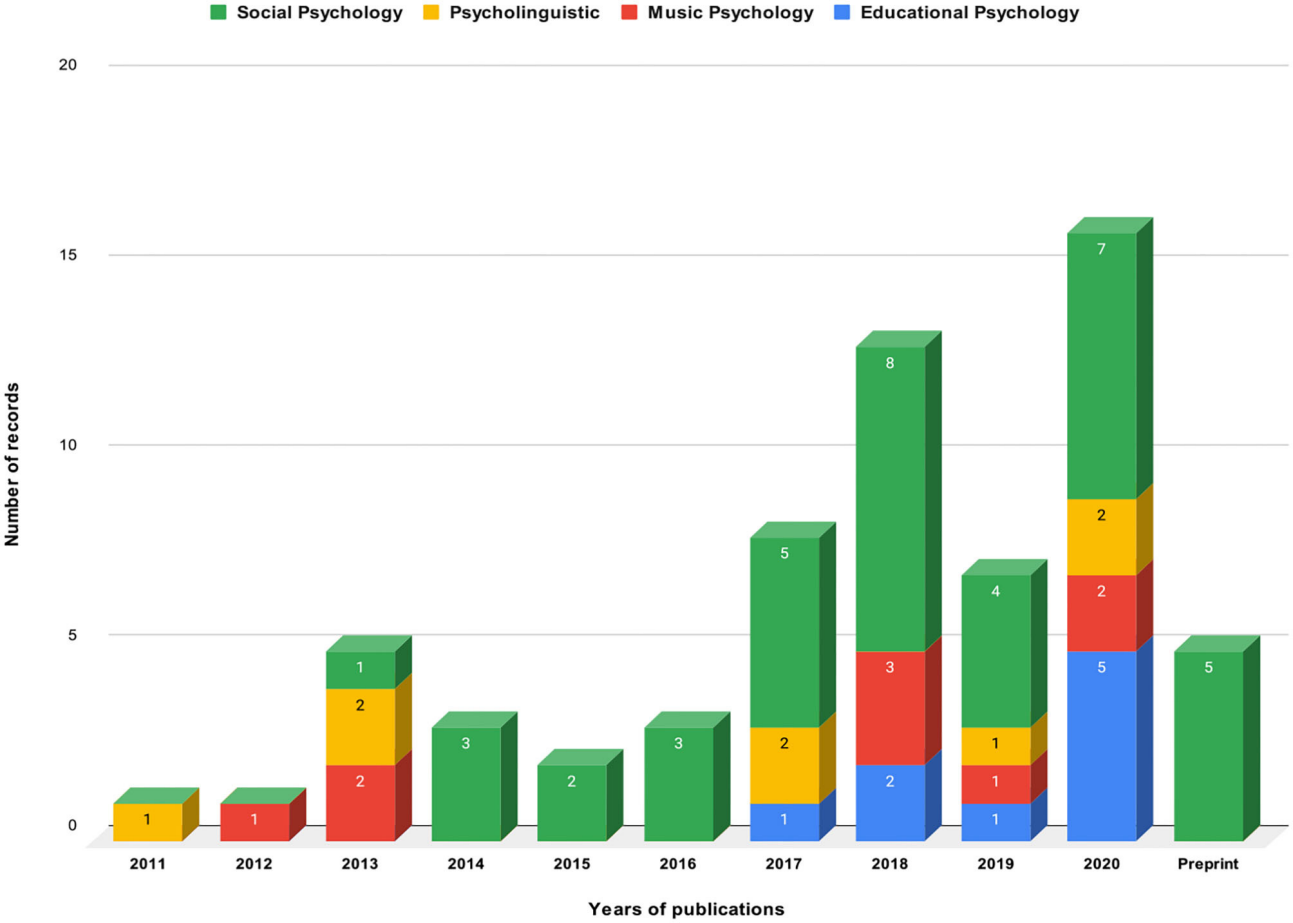
A number of studies published per year per domain.

The trend in the publication shows that there has been a surge in the number of papers published per year since 2017. In a nascent field such as this, this trend merely serves to justify the hypothesis that an increasing number of domains in psychology will be explored in terms of social dynamics and interbrain synchrony in the upcoming years. Among 64 papers, 18 or more than one-fourth of the papers were published in 2020 while four of the selected preprints were submitted in the same year. Figure 3 contains the chart showing the trends in publication grouped by their respective experimental paradigm.

### 3.3. Participants

In this systematic review, 64 articles have been screened. In the screening criteria, we include the studies with healthy participants. We excluded studies with clinical patients so that we can have a general understanding of human behavioral interaction. For instance, Dikker et al. used 10 high school students at the age of 17 for evaluating the factors that affect neural synchrony in the classroom among the students. Following Dikker et al.'s experimental paradigm, Bevilacqua et al. have demonstrated the student-teacher relationship among the 13 high school students in a biology class. The mean age of the student is 17.5 years. Besides, we have found studies showing the interaction between mother and child, the cognitive behavior while playing piano or any music, infant's interactive behavior, etc. We have mentioned the number of participants, their age, and the category of the participants in each of these 64 studies on our website aimsl.uiu.ac.bd.

### 3.4. Domain

#### 3.4.1. Experimental Paradigm

Based on the conventional and pedagogical understanding of the experimental paradigm, the authors have divided the selected studies into four main domains: social psychology, educational psychology, psycholinguistics, and music psychology. The rationale for this categorization can be pinned down to these domains themselves comprising experimental setups pre-designed by certain fine-tuned standards with theoretical backgrounds. The primary categorization of the studies was based on the experimental paradigm the studies fell under. According to Goodhew and Edwards (2019), the experimental design and setup fell under psychological paradigms. We, therefore, divided 64 studies into 4 major paradigms: educational psychology, social psychology, psycholinguistic, and music psychology. Studies were conducted in classroom settings (Dikker et al., 2017; Bevilacqua et al., 2019; Davidesco et al., 2019), while educational video watching (Poulsen et al., 2017; Cohen et al., 2018) and during teacher-student interactions were classified as educational psychology paradigm. Theoretically, this paradigm encompasses the study of how people learn, including topics such as student outcomes, the instructional process, individual differences in learning, gifted learners, and learning disabilities in laboratory settings following a set of predetermined research approaches in the field of BCI. This consisted of 14.06% or 9/64 of the studies. Theoretically, this paradigm encompasses the study of the method of learning. This includes topics such as student outcomes, the instructional process, and individual differences in learning in laboratory settings following a set of predetermined research approaches. Most studies in this paradigm had a teacher and student(s), who were set in a classroom or a classroom-like setting in a laboratory. The students were given various narrative and non-narrative technical videos (Cohen et al., 2018), audio (Dikker et al., 2017; Ding et al., 2018; Bevilacqua et al., 2019), and discussion-based stimuli. Teacher-to-student and/or student-to-student neural synchrony was measured in these studies to correlate the neural synchrony with the learning outcomes, performance, and attention measures through quizzes based on the lesson contents. Music psychology paradigm studies music performance, composition, and education under empirical experimentations. The 15.63% or 10/64 studies conducted in this domain all consisted of participants performing different instruments in duets or quartets such as guitar (Viktor et al., 2018; Fasano et al., 2020), and saxophone (Greco et al., 2018), performing choreographed dance (Poikonen et al., 2018) and enjoying music (Madsen et al., 2019; Kaneshiro et al., 2020). The performers were given different music notes to learn or perform concerning their expertise level. The melodies performed varied in terms of complexity and novelty to meet the objectives of the study. Some (Madsen et al., 2019) studies focused on the frequencies in the composed notes to study the sensory-motor association with the brain while others focused on emotion (Viktor et al., 2018) and learning methods (Fasano et al., 2020) of the performers, i.e., participants. The psycholinguistic paradigm from the BCI research follows the data collection, processing, and analysis techniques as laid out in theoretical literature by linguists and psychologists to study the psychological aspects relating to linguistic factors. The 14.06% or 9/64 of the studies selected here studied the interbrain synchrony that occurs while acquiring, using, comprehending, and producing one or more language between interlocutors. These studies consist of a speaker and listener communicating in a unidirectional (Liu et al., 2017) or bidirectional (Pérez et al., 2019) manner. Most studies are conducted in a single language, i.e., the mother language while only three explore the inter-personal synchronization in language acquisition (Reiterer et al., 2011) and comprehension (Pérez et al., 2019). The most diverse paradigm is the social psychology paradigm in which the authors grouped the rest of the studies. Social psychology studies the thoughts, feelings, and behaviors of individuals when they are influenced by the actual, imagined, and implied presence of others. This paradigm has experimented with participants who mostly have face-to-face or side-by-side interaction with each other and their conjoined behavioral analysis is measured in terms of the tasks assigned. Decision making tasks (Jahng et al., 2017), certain verbal and visual cue tasks (Reindl et al., 2019), and emotion monitoring (Nummenmaa et al., 2014; Balconi and Vanutelli, 2017; Vanutelli et al., 2017; Santamaria et al., 2019), fall under this paradigm. These studies are done under similar yet divergent experimental setups each catering to its objectives. The following studies explore machine mediated neural and behavioral synchronization in real and virtual environments (Hachmeister et al., 2014; Gumilar et al., 2021). Similarly, the effect of cooperation on neural synchrony (Abe et al., 2019), meditation and monastic debates (Fenwick et al., 2019; van Vugt et al., 2020), emotional association in groups (Nummenmaa et al., 2014; Goldstein et al., 2018; Santamaria et al., 2019) are included in this paradigm. These studies do not have a single set of specified roles for the participants similar to the previous paradigm apart from the participants all being part of social groups.

#### 3.4.2. Field of Study

**Figure 4:**
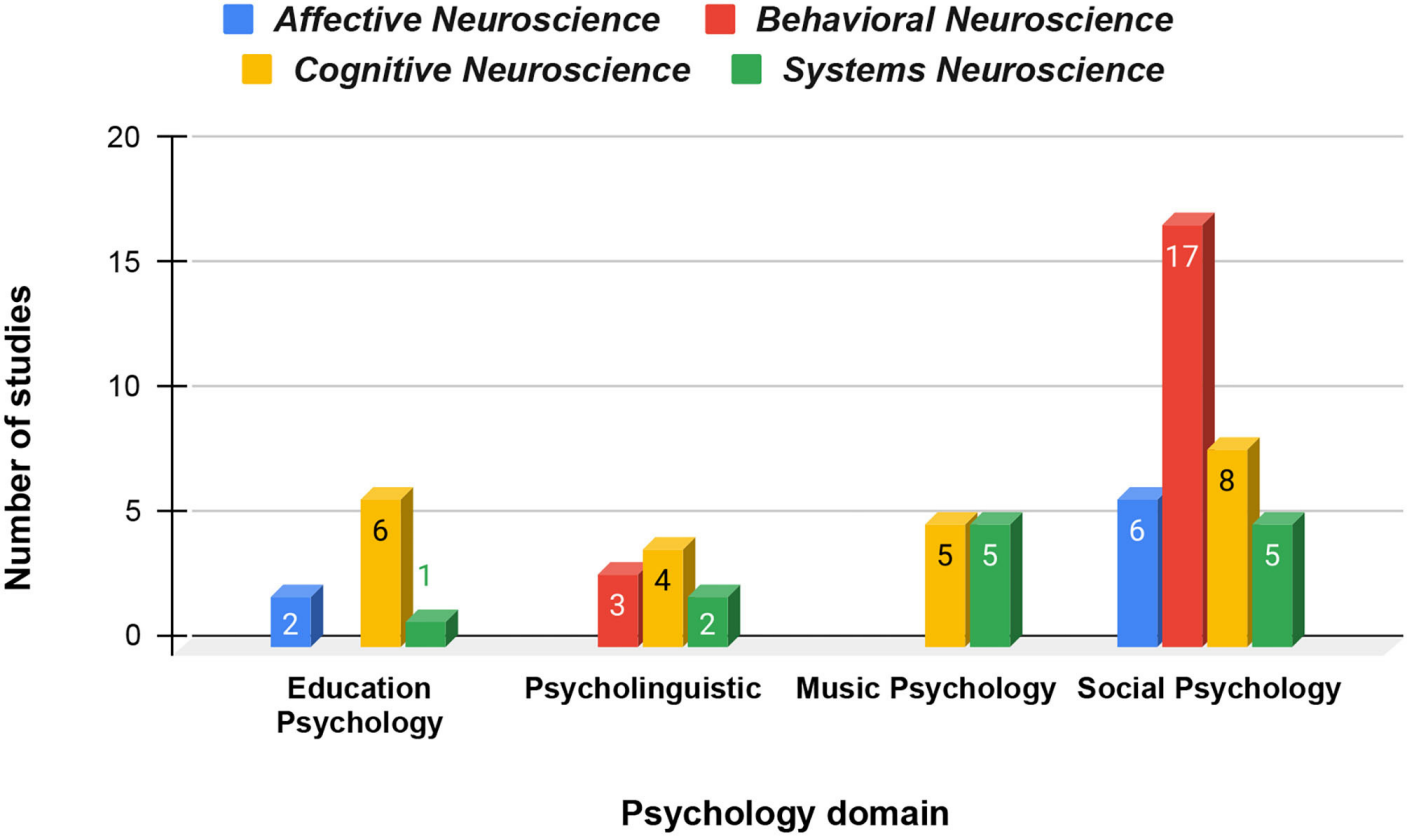
Number of fields of study under each paradigm.

To classify overlapping study contents into less ambiguous sections, the nested secondary domain classification has been done based on the focus and objectives of the selected studies. A total of 12.5% or 8/64 studies analyzing the interconnected brain dynamics of social emotions such as social connectedness (Zheng et al., 2020), empathy and emotional response to various social interactions (Ding et al., 2018) are categorized into the affective neuroscience domain. Studies that analyzed interbrain neurobiology of engagement levels (Cohen et al., 2018; Bevilacqua et al., 2019), memory retention (Piazza et al., 2020) and learning performance (Davidesco et al., 2019), and other higher-order functions of the brain are categorized into the cognitive neuroscience domain. This consisted of 36% or 23/64 of the selected studies. Behavioral neuroscience is composed of the 20 or 31.25% studies that quantified the neural underpinnings of social interactions where the participants are instructed to take part in naturalistic and semi-naturalistic exchanges as team members or opponents (Liu et al., 2016; van Vugt et al., 2020), leader or follower (Jiang et al., 2015), etc. Systems neuroscience includes the studies where the authors focus primarily on justifying the use of a new modality or development of a synchrony measure algorithm alongside exploring new signal processing and analysis pipelines (Liu et al., 2017; Viktor et al., 2018; Zamm et al., 2018) and has 20.31% or 13/64 papers in this category that has been represented in Figure 4.

#### 3.4.3. Environment

The environment of the study refers to the ecological settings in which the studies were conducted. The amount of interaction the experimenter had with the participants, the restrictions on natural movement, the laboratory's electromagnetic and sound shielding conditions, and lighting concerning the assigned tasks were the factors taken into consideration while dividing the studies into naturalistic, semi-naturalistic, and laboratory environment categories. Studies that put no restrictions on the participants regarding movement and used wireless or comparatively lighter data acquisition device was considered naturalistic and comprised 59.3% or 38/64 of the papers (Reiterer et al., 2011; Ding et al., 2018; Greco et al., 2018). Some studies restricted the movement of the participants (Dikker et al., 2017; Bevilacqua et al., 2019) through instructions, monitored ambient lighting (Fenwick et al., 2019), used noise-canceling earphones (Balconi, 2016) to limit perceived noise. These were considered semi-naturalistic as they do not replicate real-life social interactions on a full scale. The majority of the laboratory settings corresponded to fMRI settings, where the head movements were restricted to < 2– < 3 mm, and most stimuli were provided through angled mirrors or MRI-compatible headphones while the participants lay still (Fasano et al., 2020). The laboratories are electro-magnetically shielded, soundproof, and have dim lighting.

#### 3.4.4. Tasks

Most tasks in the studies were designed to carry out participatory actions for two or more participants to simultaneously evaluate the neural oscillatory dynamics associated with them. Most common tasks include cooperation-competition games or tasks (15.63% or 10/64), verbal or motor interaction (15.63%), playing musical instruments in duet or quartet (10%), prisoner's dilemma game (4%), visual cue/target task (7%), etc. A small number (9.3% or 6/64) of the studies required the participants to interact online (5/64) and/or with a computer (3/64). The tasks in these settings included watching online videos, participating in online courses, competing in online games as groups, and against each other. Some of the tasks from the educational psychology and psycholinguistics paradigms were similar due to the participants' roles as speakers or teachers and listeners or students. Each of these tasks was administered and recorded using custom-built interfaces or software, such as PsychoPy and OpenFramework -based software for research in BCI.

### 3.5. Data Acquisition

#### 3.5.1. Modality

**Figure 5:**
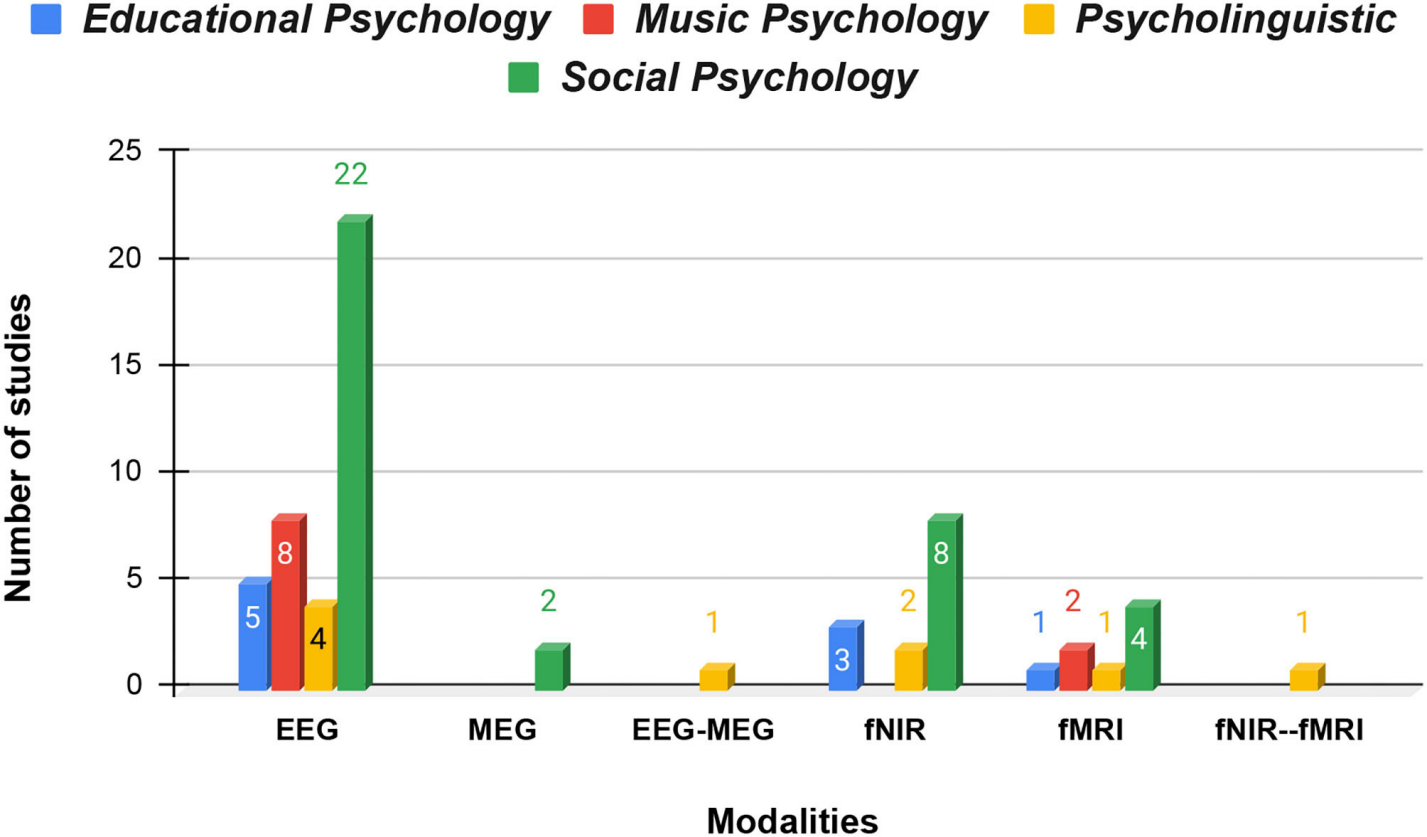
Different modalities used for the acquisition of brain signals distributed across domains.

Three modalities for data acquisition in BCI were found dominant while screening the studies. Among them, EEG was the most popular non-invasive method with 60.9% or 39/64 of studies using it for their experiments. fNIRS followed the lead with 20.3% or 13/64 studies and fMRI with 10.9% or 7/64 studies. MEG or EEG-MEG was the least exclusively used device or method for data extraction from the brain with only 3 studies. An fNIRS-fMRI-based study was also included where the authors focused on comparing the two methods before settling on fNIRS. A total of 4 studies utilized T1-weighted MRI to localize the region of interest before proceeding to apply less spatially robust modalities such as fNIRS and EEG. In studies where the analysis of the rest/active cycle of the participants was required, the researchers used Actigraphy in company with neurophysiological monitoring methods. These multi-modal studies comprised 4.7 or 3/64 of the study. Figure 5 shows different modalities used for neural signal acquisition distributed across domains.

#### 3.5.2. Hardware

Among the studies that explicitly mentioned the hardware used in BCI, BrainAmp from Brain Products took the lead (33% or 13/39) in EEG data acquisition followed by MAGNETOM Skyra 3T from Siemens for fMRI (62.5% or 5/8). Among fNIRS devices, ETG-4000 and ETG-7100 from Hitachi comprised 53.8% or 7/13 of the studies. Notably, all of these are research-grade devices that cost well above $1,000 per unit. The only consumer-grade devices included in the studies were EMOTIV EPOC and SMARITNG mBRAIN Train.

### 3.6. Analysis

The different methods and procedures followed in brain-to-brain synchrony analysis in the selected papers are as follows.

#### 3.6.1. Preprocessing

Brain signal, being contaminated with motion artifact, muscle artifact (EMG), eye artifact (EOG), and cardiac artifact (ECG), requires extensive preprocessing before being proceeded to the next step of the analysis pipeline. The preprocessing steps are applied to individual participants' data and vary widely from modality to modality. In the case of EEG, the studies used bandpass filters to filter the data within the frequency of interest ranging between 0.05 and 90 Hz. The most preferred filter was the Butterworth filter (64% or 25/39). Thresholding (67% or 26/39) and zero value replacement for signals that fell 2 to 3 SD above or below the average have also been observed to be a popular practice. Most notably, the Independent Component Analysis (ICA) was the most predominant algorithm (90% or 35/39) with regression analysis being used in the rest of the studies for EOG removal. fNIRS signals, being comparatively robust to noise, required relatively fewer preprocessing steps. The modified Beer-Lambert law was used in all the studies to convert the light intensity changes to changes in oxygenated and deoxygenated hemoglobin. Most studies preferred the study of the changes in oxygenated hemoglobin based on the evidence of correlation found in Liu et al. (2016) across multiple cognitive tasks. The signals are usually bandpass filtered in the range 0.01–0.5 Hz to focus on the frequency of interest (FOI) and eliminate cardiac oscillations. fMRI studies eliminated participants with excessive head movement <2–3 mm. Being the constituent of two different data acquisition methods is practically a 4D dataset consisting of a 3D image and temporal data. Most of the studies motion-corrected the data for head movement, performed slice-time correction, spatially transformed into standard stereotaxic spaces following the Montreal Neurologic Institute (MNI) coordinate system, and smoothed with Gaussian Kernel. The FMRIB Software Library abbreviated FSL, and Statistical Parametric Mapping Software abbreviated SPM8 or SPM12 were used in the preprocessing of the fMRI datasets.

#### 3.6.2. Neural Synchrony Measures

The studies primarily performed coupling or connectivity measures like the phase-locked value (PLV) and wavelet transform coherence (WTC) or the correlation and dependence analysis measures like the Correlated Component Analysis (CorrCA), Circular Correlation, etc. The most common measures taken by the selected studies to analyze interbrain synchronization were PLV in 16 studies, WTC in 11 studies, Intersubject Synchrony (ISC) in 6 studies, and others in the rest of the studies. To determine the directionality of information flow, the Granger Causality measure was used in 5 studies. A total of 6 other studies used Graph Theory to determine the hyper-brain network.

Here, PLV, PLI, and WTC are mostly used for connectivity analysis of neural signals among subjects for measuring brain-to-brain synchrony. While the authors need to analyze the correlation among the subjects, they have used circular correlation, correlation component analysis, General Linear Model, and find out the interbrain synchrony. Graph theory and hyper-brain networks are discussed in the articles that are focused on explaining or analyzing the directionality of information flow. Further details of these techniques for brain-to-brain analysis will be discussed in the discussion section of this paper.

### 3.7. Control Condition

Spurious synchronization between brain signals appears when the oscillations are driven by external influence, i.e., lower-level shared response to similar stimuli or in the presence of coincidental phase relationship between measured signals. Some studies acknowledged this (52 or 82%) while others did not have mention of any such phenomena. Among those mentioning induced synchrony, only 36 had implemented different experimental conditions, the majority of them implementing random or shuffled pair analysis (46 or 72%) to acknowledge fallacious synchronization due to the exposure to a shared environment.

## 4. Discussion

In this section, we review and analyze the highlights of findings of the results section and discuss the rationale with the impact of the various trends discussed above. We focus on the objectives of this review article while providing recommendations and presenting a checklist for future researchers willing to work in the field of brain-to-brain synchrony.

### 4.1. Fields of Study and Outcome in the Study of Interbrain Synchrony

As the study of brain-to-brain synchrony is a focus of neuroscientists, data scientists, and psychologists alike, the task of categorizing selected studies into domains remains one of the most complicated and perplexing tasks due to the overlapping nature of the study contents. To solve this dilemma, the authors took a pedagogical approach toward categorization so that the result can be justified using academic texts and definitions. The primary categorization of the studies was based on the experimental paradigm the studies fell under. According to Goodhew and Edwards (2019), the experimental design and setup fell under psychological paradigms. We, therefore, divided 60 studies into 4 major paradigms: educational psychology, social psychology, psycholinguistic, and music psychology. In the educational psychology paradigm, we included the studies conducted in classroom settings while students and teachers are interacting with each other (Dikker et al., 2017; Poulsen et al., 2017; Cohen et al., 2018; Bevilacqua et al., 2019; Davidesco et al., 2019). Theoretically, this paradigm encompasses the study of how people learn, including topics such as student outcomes, the instructional process, individual differences in learning, gifted learners, and learning disabilities in laboratory settings following a set of predetermined research approaches. Social psychology studies the thoughts, feelings, and behaviors of individuals when they are influenced by the actual, imagined, and implied presence of others. This paradigm has experimented with participants who mostly have face-to-face or side-by-side interaction with each other and their conjoined behavioral analysis is measured in terms of the tasks assigned. Decision making tasks (Jahng et al., 2017), certain verbal and visual cue task (Reindl et al., 2019), and emotion monitoring (Nummenmaa et al., 2014; Balconi and Vanutelli, 2017; Vanutelli et al., 2017; Santamaria et al., 2019) fall under this paradigm.

The secondary classification was done based on the focus of the study of the selected BCI papers. This converged on the neuroscience field since most of the measured functionalities of the brains of the participants and the analyses that followed were parts of different neuroscience domains. Papers that measured engagement levels, memory retention, and learning performance are categorized into the cognitive neuroscience domain. Studies analyzing the brain dynamics of different social emotions such as social connectedness, empathy, and emotional response to different social interactions are part of the affective neuroscience domain. Behavioral neuroscience comprises the studies that quantified the neural underpinnings of social interactions where the participants are instructed to take part in naturalistic and semi-naturalistic exchanges. Systems neuroscience includes the studies where the authors focus primarily on justifying the use of a new modality, extracting a specific frequency band or a cortical or deep brain region concerning a task, or develop a new synchrony analysis measure.

### 4.2. Data Acquisition Modalities Used in Brain-to-Brain Synchrony Studies

We are indebted to the large variety of methods developed to measure brain activity in the late-twentieth and twenty-first centuries. Based on the neural signals' spatial and temporal resolution and the portability of the device using BCI technology, the most popular ways to measure the interbrain synchrony are EEG, fMRI, and fNIRS. By analyzing the modalities used in a group of studies, we can further understand different behavioral functions and their neural underpinnings. As we found in the results, most of the studies here were conducted using EEG devices. The reason behind this was due to EEG's ability to provide a naturalistic setting for the experiments (Jahng et al., 2017; Cohen et al., 2018; Kaneshiro et al., 2020). Unlike fMRI and fNIRS, EEG measures neural activity directly from the electrical currents through electrodes placed on the scalp. This enables EEG to record brain signal changes on a millisecond scale, making it a very strong candidate for research involving temporally synchronized social interactions (Kaneshiro et al., 2020). The relatively low price has made it very convenient for researchers to measure a large number of scalp activities at once as demonstrated by Dikker et al. (2020). However, EEG is susceptible to muscle movement artifacts as well as a considerable amount of ocular artifacts. Moreover, EEG suffers from volume conduction effect, making it difficult to correlate to the specific brain areas of the participants. Table 5 shows a list of studies that utilized EEG as their neural signal acquisition modality in different psychological experimental paradigms. As opposed to EEG, MEG provides high-resolution spatiotemporal dynamics of neuromagnetic fields during various cognitive and behavioral activities, making it a great candidate for brain-to-brain synchrony analysis. Despite being impractical in terms of mobility, fMRI has been the first device used in a hyperscanning study (Montague, 2002). It is a method that indirectly measures brain activity by detecting changes associated with blood flow, which is the blood-oxygen-level-dependent (BOLD) contrast. BOLD contrast enables researchers to study deep brain structures noninvasively while also compromising temporal resolution. However, fMRI is capable of capturing data from the default mode network (DMN) which is an impossible feat for EEG. Due to the immobility and high cost of the experiment setups, very few studies have been conducted with the fMRI method (Nummenmaa et al., 2014; Gao et al., 2020). Apart from this, the complexity of the fMRI data requires the development of new analysis models which further hinders the designing of experimental setups. However, this can be of great value if combined with EEG to compensate for its temporal resolution as demonstrated by Koike et al. (2016). One of the most recent inventions in the field of neuroimaging is fNIRS. It is also a passive method that uses the contrast between oxygenated and deoxygenated hemoglobin in the brain to measure the changes in the superficial brain regions with a low spatial resolution and a comparatively low temporal resolution. But due to its resistance to motion artifacts and its mobility, it is widely used in the field of hyperscanning. Nozawa et al. (2016) utilized fNIRS to study group communications in a naturalistic setting with four participants. The portability of fNIRS also makes it a suitable choice for investigating the brain functions of toddlers. Reindl et al. (2018) showed in their study the relationship between emotion regulation in different circumstances and the brain-to-brain synchrony in parent-child dyads. Liu et al. (2016) investigates brain-to-brain coupling between speakers and listeners to analyze the neural correlates of verbal communication across the group using both fNIRS and fMRI (Liu and Pelowski, 2014). By comparing optodes and voxels from the same brain regions, they concluded that listeners' brain activity significantly correlates with speakers with a delay and disappears when verbal communication fails. A similar study was carried out with EEG-MEG where the delay in speaker -listener synchrony was also accounted for using the high spatiotemporal resolution of the two devices combined.

**Table 5:** Selected studies on the dimensions of this review.

**Paradigm**	**Field of study**	**Experimental setting**	**Modality**	**Synchrony measure**	**References**
Educational psychology	Affective neuroscience	Naturalistic	fNIRS, Actigraph	Wavelet transform coherence	Pan et al. (2020b)
		Laboratory	fNIRS	Wavelet transform coherence	Zheng et al. (2020)
				Granger causality	Zheng et al. (2020)
	Cognitive neuroscience	Naturalistic	EEG	Correlated component analysis	Poulsen et al. (2017); Cohen et al. (2018)
		Semi-naturalistic	EEG	Total interdependence	Dikker et al. (2017); Bevilacqua et al. (2019)
				Circular Correlation	Davidesco et al. (2019)
		Laboratory	fMRI	Inter-subject Correlation	Nguyen et al. (2020a)
	Systems neuroscience	Naturalistic	fNIRS	Wavelet transform coherence	Pan et al. (2020a)
Music psychology	Cognitive neuroscience	Naturalistic	EEG	Correlated component analysis	Madsen et al. (2019)
				Inter-subject correlation	Kaneshiro et al. (2020)
				Phase synchronization index	Poikonen et al. (2018)
		Laboratory	fMRI	Pearson correlation	Abrams et al. (2013)
				Inter-subject correlation	Fasano et al. (2020)
	Systems neuroscience	Naturalistic	EEG	Graph theory	Müller et al. (2013); Greco et al. (2018)
				Interbrain Phase Coherence	Sänger et al. (2012)
				Phase locking index	Sänger et al. (2012)
				Phase synchrony index	Sänger et al. (2012); Müller et al. (2013); Greco et al. (2018)
		Semi-naturalistic	EEG	Graph theory	Viktor et al. (2018)
		Laboratory	EEG	Amplitude envelope correlation	Zamm et al. (2018)
Psycholinguistics	Behavioral neuroscience	Naturalistic	EEG	Partial directed coherence	Leong et al. (2017)
				Phase-locking value	Leong et al. (2017)
				Granger causality	Leong et al. (2017)
			fNIRS	Wavelet transform coherence	Nguyen et al. (2020c)
		Laboratory	EEG-MEG	Phase lag index	Ahn et al. (2018)
	Cognitive neuroscience	Naturalistic	EEG	Circular correlation	Pérez et al. (2019)
		Semi-naturalistic	EEG	Cross correlation analysis	Kawasaki et al. (2013)
			fNIRS	Inter-subject correlation	Piazza et al. (2020)
		Laboratory	EEG	Coarse-graining markov-chain	Reiterer et al. (2011)
				Phase-lag index	Reiterer et al. (2011)
	Systems neuroscience	Laboratory	fNIRS-fMRI	General linear model	Liu et al. (2017)
			fMRI	Inter-subject correlation	Dikker et al. (2014)
Social psychology	Affective neuroscience	Naturalistic	EEG	Circular correlation	Goldstein et al. (2018)
				Inter-subject correlation	Ding et al. (2018)
				Pearson correlation	Dikker et al. (2019)
				Phase-locking value	Ding et al. (2018); Zhu et al. (2018)
				Spearman correlation	Kinreich et al. (2017)
		Laboratory	fMRI	Instantaneous intersubject phase synchronization	Nummenmaa et al. (2014)
	Behavioral neuroscience	Naturalistic	EEG	Partial correlation	Szymanski et al. (2017); Balconi et al. (2018)
				Partial directed coherence	Santamaria et al. (2019); Shehata et al. (2020)
				Phase-locking value	Jahng et al. (2017); Mu et al. (2017); Santamaria et al. (2019)
					Yun et al. (2012); Shehata et al. (2020)
				Phase lag index	Szymanski et al. (2017)
				Granger-gewek causality	Shehata et al. (2020)
				Graph theory	Santamaria et al. (2019)
			fNIRS	Wavelet transform coherence	Jiang et al. (2015); Hu et al. (2017); Zhang et al. (2018)
				Granger causality	Jiang et al. (2015)
		Semi-naturalistic	EEG	Phase-locking value	Hu et al. (2018)
		Laboratory	EEG	Phase synchronization index	Kawasaki et al. (2018)
			MEG	Phase-locking value	Hirata et al. (2014); Zhou et al. (2016)
				Granger causality	Hirata et al. (2014)
				Partial directed coherence	Hirata et al. (2014)
			fNIRS	Wavelet transform coherence	Reindl et al. (2018)
			fMRI	Correlation component analysis	Koike et al. (2016); Abe et al. (2019)
	Cognitive neuroscience	Naturalistic	EEG	Phase-locking value	Antonenko et al. (2019); van Vugt et al. (2020)
				Phase Slope Index	Fenwick et al. (2019)
				Total Interdependence	Reinero et al. (2020)
			fNIRS	Wavelet transform coherence	Liu et al. (2016)
		Semi-naturalistic	fNIRS	Wavelet transform coherence	Nozawa et al. (2016); Nguyen et al. (2020b)
		Laboratory	fNIRS	Wavelet transform coherence	Xue et al. (2018)
	Systems neuroscience	Naturalistic	EEG	Phase-locking value	Hachmeister et al. (2014); Barraza et al. (2020); Gumilar et al. (2021)
		Laboratory	EEG	Circular correlation	Novembre et al. (2017)
			fMRI	Inter-subject correlation	Gao et al. (2020)

### 4.3. Brain-to-Brain Synchrony Analysis Measures

The synchrony measures are estimators of synchrony between more than one neural signal in continuous time series where lower values indicate independent time series and higher values stand for various degrees of correlation. The methods previously used to study single brain, i.e., intra-brain connectivity are now adapted and thus the most common methods to estimate the strength of neural connectivity between multiple brains. In the selected studies, various connectivity, correlation, and direction measures were taken to determine the synchrony between two or more brains.

#### 4.3.1. Connectivity Measures

The majority of the connectivity methods are based on second-order measures in the frequency domain. This is dependent on the principle that two electrodes from two different brains being in coherence denote functional synchronization. Methods like the phase-locking value (PLV), phase coherence, and phase lag index (PLI) were previously used to measure intra-brain synchronization. PLV, as the name suggests, is a measure to determine the phase-locked state of two signals within a given time-window following the Fourier Transform of the said signal. PLV being 1 denotes the observed phases being in perfect synchrony in a particular frequency and 0 denotes complete asynchrony. This measure was used in most of the EEG interbrain synchrony studies. They investigated cortical synchronization while two participants tried to imitate their hand (Dumas et al., 2011) or finger movements (Yun et al., 2012) during a coordinated time estimation and speaking and listening (Pérez et al., 2019) and during a cooperative decision-making task (Hu et al., 2017). Another similar measure, PLI, was used in studies investigating coordinated behavior in guitar players playing in duets (Sänger et al., 2012). Despite their similarities, PLI is not susceptible to common source problem as PLV (Aydore et al., 2013). However, for multiple brain research, having sources in different brains, results from these measures are similar. Phase coherence, a similar method of investigating synchronization within or between brains, is also dependent on neural oscillations' phase. This method was used in multiple variations across the selected studies. Notable among them are the studies mentioned above that investigated guitar players (Sänger et al., 2012; Viktor et al., 2018). The major benefits of phase synchronization over other coherence measures are its better time resolution and sensitivity to phase, rather than amplitude. The property of being restricted to a frequency band has led to its use in specific EEG bands reflecting cognitive process and attention states. Wavelet transform coherence (WTC) is a pertinent method to measure the coherence of two signals. As a modified version of Fourier Transform coherence, WTC was developed to analyze the geophysical time series at the beginning of this century. However, it found its application in neuroscience, especially in analyzing fNIRS studies. Being the only method used to analyze interbrain connectivity with fNIRS, it is also one of the most common analysis methods within all selected studies. WTC was used to estimate interbrain synchrony in all paradigms to study action monitoring, cooperative and competitive behaviors (Liu et al., 2016; Cheng et al., 2019), communication (Jiang et al., 2015; Nozawa et al., 2016) and teaching/learning behaviors (Pan et al., 2020b). All connectivity measures mentioned in this section are interpreted as synchrony between brains in the studies and are almost always referred to as interbrain synchrony, while in reality, it infers the interbrain or inter-electrode functional connectivity.

#### 4.3.2. Correlation Analysis

The estimation of correlation coming from different brains or the correlation between behavioral synchrony and neural connectivity is yet another synchrony measure. Within the selected studies, data collected from all modalities, i.e., EEG, fNIRS, and fMRI were subjected to correlation measures. The low temporal resolution of fMRI prevented the researchers from analyzing the higher frequency ranges which are responsible for higher cognitive function of the mammalian brain. Therefore, linear dependence was used to measure the similarities in oscillation between two brains. Additionally, the BOLD signal itself was used for correlation analysis, but regression model coefficients derived mostly from General Linear Model were represented as activations in different tasks. These types of analyses were applied in research investigating mutual gaze, shared attention, and cooperation in the joint force production task (Koike et al., 2016; Abe et al., 2019). Neural synchronization between two or more brains was estimated from the correlation found in these studies. Correlation measures were also applied to EEG neural synchrony data. Moreover, different aspects of EEG signals were used for correlation analysis. Correlation between different frequencies (theta and alpha) was used to investigate the coordination and comprehension of speech rhythm (Kawasaki et al., 2013) and differences between interactions between strangers and couples in alpha, beta, and gamma (Kinreich et al., 2017). Furthermore, the total interdependence analysis was used in a study that investigated brain synchronization in a naturalistic classroom environment on a group of students (Dikker et al., 2017; Bevilacqua et al., 2019). This analysis was used to predict classroom dynamics and engagement between students and teachers under different teaching conditions. Lastly, two fNIRS experiments applied correlation analysis to estimate synchrony between brains in tasks that required cooperation or competition between participants (Liu et al., 2016; Fasano et al., 2020). In Table 6, the characteristics of synchrony have been presented relating the cases with different methods for synchrony measurement.

**Table 6:** Characteristics of different synchrony measures.

**Case**	**Coherence**	**Wavelet**	**Phase-**	**Granger**	**Partial**
		**Transform**	**locking**	**causality**	**directed**
		**coherence**	**value**		**coherence**
Linear	X	X			
Non-linear			X		
Info-based				X	X
Data-driven	X	X	X	X	X
Causality assessing				X	X
Multivariate					X
Stationary independent		X	X		
Functional connectivity	X	X	X	X	X
Effective connectivity				X	X

#### 4.3.3. Graph Theory and Hyperbrain Networks

In brain-computer interface, brain networks are represented as complex systems using graph theory methods on connectivity matrices from neural signals. The structure of an adjacency matrix of a graph is similar to the nodes and edges of the brain network. Graph theory measures focus on different parameters of intra- and inter-brain networks. The use of graph theory allowed the researchers to explore the hyperbrain network, a complex intra- and inter-brain network model at different granularity levels. From the selected studies, the only studies utilizing this were the studies in the music psychology paradigm. Different graph theory measures were used to study the relationship between small-worldness and modularity in hyperbrain networks in guitar players. Small-worldness of interbrain networks was enhanced during musical coordination (Sänger et al., 2012), and the topology of the networks was more prominent in higher frequencies than in lower (Viktor et al., 2018). The strength and importance of the links in the network can determine the level of effect an interactive action has on the interconnected brains and to which extent these connections can enhance collective performance.

#### 4.3.4. Information Flow

To study the driver-response relationship between interlocutors, analysis of the flow of information is a crucial factor. Additionally, definite proof of causality also strengthens the mechanistic hypothesis of interbrain synchrony. This method requires the establishment of a causal link between the measured brains. Granger Causality and Partial Directed Coherence (PDC) are such methods that meet this criterion. In the selected studies, the flow of information was utilized to measure the direction of information flow in teacher-student relationship (Nguyen et al., 2020a), leader-follower relationship (Jiang et al., 2015), and meditation trainer, and follower interbrain synchrony. As hypothesized, the flow of information was controlled by the leading personnel in the dyads.

In most cases, the advantage and disadvantages of these empirical measures are not highlighted due to the novelty of the approaches. The current standard is based on intra-brain synchrony measures and is yet to be fully analyzed and exploited in interbrain synchrony analysis. Furthermore, as a growing number of measures are being used as exploratory techniques, it is yet to be simulated whether they relate to the same set of neurophysiological processes.

### 4.4. Control Conditions Discussed in the Selected Papers

Most studies in this field are based on the conjecture that the perceived brain-to-brain synchrony is the direct consequence of information flow between two interacting brains. In reality, two different perspectives are taken into account while interpreting synchronized oscillations. The mechanistic approach corroborates the generalized and widely accepted principle that interbrain synchrony facilitates inter-personal information flow. Hence, this allows the associated individuals to enhance their social performance. However, the epiphenomenal stance takes into account two cases: induced synchrony, or the result of a shared perception of a low-level stimulus and coincidental phase relationship between individual rhythms, or simply, coincidental synchrony. The latter perspective states that induced or coincidental synchrony has no role in determining causality in social behavior. Although credit is still due when providing correlational evidence, most hyperscanning experimental setups do not differentiate between correlational and causal evidence. Primarily, as a control for coincidental synchrony, experimental conditions remain identical in every way possible, except for one where participants are socially engaged and another when they are not (Burgess, 2013). This is implemented through random pair analysis followed by 1,000 or more iterated permutation analysis which is then Bonferroni corrected. As a result, only the emergent neural synchronization due to real interaction survives the analysis. While many other control experiments remain prevalent, including scrambled stimuli (Nguyen et al., 2020c) and multivariate regression of gyroscopic head and eye movement (Dikker et al., 2017), the most effective is a methodological approach, namely, Granger Causality (Leong et al., 2017; Zheng et al., 2020). In more recent years, an additional interventional approach has been proposed (Hoehl et al., 2020) to render the analytical approach even more robust and well-grounded. A non-invasive multibrain stimulation has been proposed to reverse-engineer the conventional interaction vs. synchrony relationship in the field of BCI. Under the influence of the directed stimulus, interbrain synchrony can be manipulated and act as the independent variable while the resultant social interactions are being studied. The only single study among the selected others to employ this has been (Novembre et al., 2017), which demonstrated the effect of the beta band (20 Hz) in-phase currents (hyper-tACS) in the motor cortex in determining the performance enhancement of participants' finger-tapping task. This multibrain stimulation (MBS) approach may allow a paradigm shift in the coming years.

## 5. Brain-to-Brain Synchrony in the Development of the BCI

Brain-computer interface is an emerging field of research with various applications. The contribution of BCI has been reached from mind-reading to remote communication and control in numerous research and real-life applications such as human behavioral interaction acknowledgment, cognitive behavior analysis, education, self-regulation, neuromarketing, and understanding of the dynamics of social interaction that creates mutual interaction. Measuring neural synchrony among two or more subjects gives us the opportunity to quantify neural engagement and collaboration for improving attention, focus, and productivity that can be achieved through the Neurofeedback loop. Perez et al. measured the brain-to-brain synchrony of subjects while speaking and listening to native and foreign languages (Pérez et al., 2019). This study shows the cognitive-behavioral perspective of human beings while speaking or listening to their mother tongue or other languages. They used circular correlation to determine interbrain synchrony between the subjects. There is a great number of research ongoing in the education domain where authors are using different brain-to-brain synchrony techniques to understand the factors that affect students' focus or attention in the class or neural synchrony between teacher and students (Dikker et al., 2017, 2019; Davidesco et al., 2019). These help us to build an efficient educational system. Our systematic review has focused on 64 significant studies that are using different neural synchrony measure techniques for understanding educational neuroscience, social psychology, cognitive behavior, and behavioral neuroscience. The authors conducted a multi-step categorization of the selected studies for their coinciding presence in multidisciplinary domains of neuroscience and psychology where neural synchrony has been measured. This systematic review provides a comprehensive analysis of techniques, methodology, and translational algorithms for evaluating brain-to-brain in BCI research.

## 6. Limitation and Recommendation

The authors are optimistic regarding the contribution of this transparent and reproducible systematic review's capability to assist future researchers working on the connectivity of the human brain as it has explored almost every discipline of neuroscience and psychology that contains the functional connectivity study of brain-to-brain synchrony in BCI research. Therefore, the authors found it within their responsibility to note down the challenges and suggestions from this systematic review of brain-to-brain synchrony studies as follows:

- To complement a large number of correlational studies on brain-to-brain synchrony, more experimental and quasi-experimental studies should be conducted with dependent and independent variables in a controlled environment to test causation.
- A wide range of social activities is yet to be experimented on, such as family affiliations, popular entertainment perception, military drills, mob mentality, etc. These provide extensive scope for future researchers.
- To successfully utilize low-cost consumer-grade EEG in naturalistic settings, the EEG inverse problem can be used to localize the source more precisely than the original spatial resolution EEG offers. This will allow a full transition into the naturalistic environment.
- While developing or adapting new synchrony measures emphasis should be given to preserving temporal information while computing functional connectivity to increase time resolution. This is necessary when the activities have very short-lasting stimuli and the underlying neural dynamics are fast-changing.
- Various neural recording and stimulus methods such as hyper-tACS are still required to reverse engineer and manipulate inter-brain synchrony to study the prolonged effects on adapted social interactions.
- Studies can be conducted on modulating inter-brain synchrony with various interventional methods to increase inter-personal empathy or as a therapeutic measure for psychological disorders such as antisocial personality disorder and social anxiety in the BCI and neurofeedback settings.

## 7. Conclusion

To date, brain-to-brain synchrony analysis is a relatively untapped opportunity to study and unveil the brain dynamics of human interaction as a social being in the field of BCI. Measuring brain-to-brain synchrony is an endeavor to discover human individualities vs. team behaviors and the reason behind these behaviors is by exploring neural activity during shared actions and experiences. The leading purpose of this systematic review was to analyze the state of sharing features and attributes among a group of participants and examine their neural synchronization. Authors have established that over the progression of the last 10 years, most of the studies of brain-to-brain synchrony were associated with cooperation tasks practiced through games, social interactivity, and studied the notable evolution of the educational paradigm. The evolutionary breakthrough of this review was the successful categorization of the complex overlap of the scopes of the studies that have been separated into forms of neuroscience and psychological domains for each study. A total of 86% of the studies focused on quantifying the correlation between shared tasks and their associated synchrony index while the rest of the experimental and quasi-experimental studies focused on the development of new processing pipelines and empirical evidence of multi-modal approaches in measuring brain-to-brain synchrony. EEG, being the most used neural recording technique, paved a way for more naturalistic studies while also being responsible for the development of many time-resolved and frequency-specific measures and algorithms. A large number of intra-brain synchrony measures were adapted to perform in the interbrain studies, along with a smaller number of directional and network models. Apart from mimicking a truly ecological set up, the largest challenge to date is the lack of exploitation of tools and methods in obtaining causal evidence and the distinction between mechanistic and epiphenomenal events. Reviewing the studies on brain-to-brain synchrony has paved the way to understanding the future scopes and optimal practices in this area.

## Data Availability Statement

The original contributions presented in the study are publicly available. This data can be found here: https://sites.google.com/view/brain-synchrony-review/home.

## Author Contributions

KM: conceptualization, methodology, validation, and project administration. TC, RV, MA, MS, and WJ: validation and review. II and TN: investigation, data curation, draft, and review and editing. All authors contributed to the article and approved the submitted version.

## Funding

The authors would like to express their gratitude to ICT Innovation Fund from ICT Division, Ministry of Posts, Telecommunications, and Information Technology, People's Republic of Bangladesh, for their funding (Code no: 1280101-120008431-3631108) and continuous support in the research and development of this project.

## Conflict of Interest

WJ was employed by BrainCo Inc. The remaining authors declare that the research was conducted in the absence of any commercial or financial relationships that could be construed as a potential conflict of interest.

## Publisher's Note

All claims expressed in this article are solely those of the authors and do not necessarily represent those of their affiliated organizations, or those of the publisher, the editors and the reviewers. Any product that may be evaluated in this article, or claim that may be made by its manufacturer, is not guaranteed or endorsed by the publisher.
